# *Valeriana officinalis* Counteracts Rotenone Effects on Spreading Depression in the Rat Brain *in vivo* and Protects Against Rotenone Cytotoxicity Toward Rat Glioma C6 Cells *in vitro*

**DOI:** 10.3389/fnins.2020.00759

**Published:** 2020-07-23

**Authors:** Ana Paula Amaral de Brito, Isabel Michely da Silva Galvão de Melo, Ramon Santos El-Bachá, Rubem Carlos Araújo Guedes

**Affiliations:** ^1^Department of Biochemistry and Biophysics, Universidade Federal da Bahia, Salvador, Brazil; ^2^Department of Nutrition, Universidade Federal de Pernambuco, Recife, Brazil

**Keywords:** rat, *Valeriana officinalis*, cortical spreading depression, rotenone, brain excitability, toxic effects, astrocyte

## Abstract

Astrocytes can protect neurons against oxidative stress and excitability-dependent disorders, such as epilepsy. *Valeriana officinalis* has been used as anticonvulsant and can exert an antioxidant effect, which may underlie its opposing action against the toxic effects of the pesticide rotenone. We investigated the *V. officinalis***/**rotenone interaction in the cortical spreading depression (CSD), a phenomenon that depends upon brain excitability (*in vivo model)*. In addition, we analyzed the protective action of *V. officinalis* against the cytotoxic effects of rotenone in cultures of rat C6 glioma cells (*in vitro* model). For the CSD study, Wistar rats received either *V. officinalis* (250 mg/kg/day via gavage for 15 days; *n* = 8) or 10 mg/kg/day rotenone via subcutaneous injections for 7 days (*n* = 7), or they received both substances (*n* = 5). Two control groups received either saline (vehicle for *V.* officinalis; *n* = 8) or 1% Tween-80 aqueous solution (vehicle for rotenone; *n* = 9). After treatment, CSD was recorded for 4 h. The rotenone- and *V. officinalis*-treated groups presented, respectively, with lower (2.96 ± 0.14 mm/min), and higher CSD propagation velocity (3.81 ± 0.10 mm/min) when compared with the controls (Tween-80, 3.37 ± 0.06 mm/min and saline, 3.35 ± 0.08 mm/min; *p* < 0.05). The rotenone plus *V. officinalis*-treated group displayed a CSD velocity (3.38 ± 0.07 mm/min) that was similar to controls. In line with these results, *in vitro* experiments on rat glioma C6 cells revealed a protective effect (MTT assay) of *V. officinalis* against rotenone-induced cytotoxicity. These results suggest the therapeutic potential of *V. officinalis* for treating neurological diseases involving redox imbalance and astrocyte dysfunction.

## Introduction

Astrocytes are intimately involved in diverse neuronal functions, such as modulating synaptic activity and plasticity; regulating the extracellular microenvironment by buffering neurotransmitter, ion, and water content, and regulating local blood flow and the delivery of energy substrates (Devinsky et al., 2012). Dysregulation of astrocyte function may cause seizures or promote epileptogenesis (Wetherington et al., 2008; Chan et al., 2019).

The brain is very sensitive to oxidative stress because of its high rate of O_2_ and energy consumption, relatively low levels of antioxidants, and large amount of oxidizable unsaturated fatty acids (Coimbra-Costa et al., 2017). The combined occurrence of high utilization of energy and impairment of oxidative phosphorylation jeopardizes the cells’ capacity to regulate their energy levels. This can lead to excessive production of reactive oxygen species (ROS), causing neuronal injury. Impaired energy metabolism may play a critical role in the neuronal injury caused by oxidant substance-induced epilepsy (Gupta et al., 2000; Bolson and Steinhäuser, 2018).

The plant known as *Valeriana officinalis* L., Valerianaceae has long been used for the treatment of insomnia, and it is recognized as an effective herbal sedative worldwide. In some countries, *V. officinalis* was additionally a reasonably popular anticonvulsant remedy in the past (Gorji and Khaleghi Ghadiri, 2001; Eadie, 2004). The aqueous extract of *V. officinalis* exerts effective antiepileptic action in the model of temporal lobe epilepsy (Rezvani et al., 2010; Wu et al., 2017). It is believed that the pharmacological activity of *V. officinalis* depends on its antioxidant action, and data in the literature have pointed to a protective effect of *V. officinalis* in rotenone-induced neural toxicity (Oliveira et al., 2009; Sudati et al., 2013). Despite the fact that in those studies *V. officinalis* protected neurons against rotenone-induced cytotoxicity, the protective effect was not tested in cells of glial origin. This is important because astrocytes supposedly play a role in the loss of dopaminergic neurons in Parkinson’s disease (Nagatsu and Sawada, 2005). Our work advances the knowledge in the field by studying (to the best of our knowledge for the first time) the *V. officinalis* effects on cells of glial origin. Furthermore, we demonstrated a novel action of *V. officinalis* extract on the cortical spreading depression (CSD) phenomenon *in vivo* (see below), which also represents an advancement of the knowledge in this field. Supported by these results, the present study investigated the hypothesis that *V. officinalis* methanol extract protects cells of glial origin against rotenone-induced cytotoxicity *in vitro*.

Rotenone is an aromatic compound that is largely employed as pesticide (Jenner, 2001). Rotenone passes across cell membranes without difficulty, as it is a very lipophilic compound and does not depend upon membrane transporters (Greenamyre et al., 2001). Rotenone inhibits the mitochondrial enzyme NADH dehydrogenase, acting within complex I of the respiratory chain (Talpade et al., 2000; Thiffault et al., 2000; Pamies et al., 2018) producing free-radical-dependent oxidative injury (Chen et al., 2008). Therefore, exposing rats to rotenone constitutes an interesting and useful model for studying brain disorders that are related to redox imbalance. Indeed, studies have stressed the importance of astrocytes in the protection of neurons from rotenone (Rathinam et al., 2012; Goswami et al., 2015). Furthermore, therapeutic efforts aimed at the removal of free radicals or prevention of their formation in the brain may be beneficial in epilepsy. Natural products represent an important source of chemical compounds, as they often have potent biological activities with promising pharmacological profiles.

The *in vivo* experiments in this study investigated the electrophysiological effects of rotenone administration and the counteracting effect of treatment with *V. officinalis* on the velocity of propagation of CSD in the rat brain. CSD is a reversible response of the brain tissue to electrical, mechanical, or chemical stimuli. The response consists of cell depolarization with subsequent electrical depression, which propagates slowly over the tissue (Leão, 1944, 1947; Guedes and Abadie-Guedes, 2019). CSD has been further electrophysiologically described in many animal species (Gorji, 2001) and in humans (Dreier et al., 2019). Modifications in brain excitability (Koroleva and Bures, 1980) and in the redox homeostasis of the brain (El-Bachá et al., 1998; Mendes-da-Silva et al., 2014) are factors that modulate CSD. Analyzing some CSD features (e.g., velocity of propagation) constitutes a very useful way of evaluating brain sensitivity to CSD under conditions that can modify brain excitability (Amaral et al., 2009; Guedes and Abadie-Guedes, 2019) and therefore are clinically relevant. Certain experimental treatments can facilitate CSD propagation, whereas other treatments can antagonize CSD (Guedes and Abadie-Guedes, 2019). These treatments can help in the comprehension of the CSD mechanisms and perhaps shed light on the mechanisms of CSD-related diseases, such as epilepsy (Leão, 1944, 1972; Guedes and Cavalheiro, 1997; Guedes and Abadie-Guedes, 2019).

Extracts of medicinal plants have beneficial effects in experimental models both *in vivo* (Rezvani et al., 2010 for *V. officinalis*, and Ilesanmi et al., 2019 for *Antiaris africana*) and *in vitro* (Areiza-Mazo et al., 2018 for *Physalis peruviana*). Therefore, in addition to the CSD model, we analyzed the *V. officinalis*/rotenone interaction in a set of *in vitro* experiments in the rat C6 glioma cell culture model (Grobben et al., 2002; Krylova et al., 2019). Rat C6 glioma cells have been used to study the effects of valproic acid as an inhibitor of histone deacetylase (Ximenes et al., 2012). This short-chain fatty acid is an analog of valeric acid, which is present in *V. officinalis*. Since these cells preserve several characteristics of astrocytes and are easy to cultivate, they were used in this work to investigate the protective effect of the methanolic extract of *V. officinalis* root powder against rotenone-induced cytotoxicity.

Using the CSD and the C6 glioma cell culture models, we addressed the following two questions: first, how does the administration of rotenone or *V. officinalis* affect CSD propagation and glial cell viability in culture, and second, does *V. officinalis* protect the rat brain against rotenone-induced toxicity? Here, we provide evidence that *V. officinalis* counteracts rotenone effects on CSD in the rat brain *in vivo* and protects against rotenone cytotoxicity on the viability of rat glioma C6 cells *in vitro*.

## Materials and Methods

### *In vivo* Experiments

Male Wistar rats (2 months old; 240–270 *g* body weight; *n* = 37) were housed in polypropylene cages (51 × 35.5 × 18.5 cm) under controlled temperature (23 ± 1°C), and a 12:12 h light:dark cycle (lights on at 7:00 a.m.); animals were fed a lab chow diet containing 23% protein. All experiments were performed between 13:00 and 18:00 h. The animals were handled in accordance with the norms of the Ethics Committee for Animal Research of our University. These norms are coherent with the “Principles of Laboratory Animal Care” (NIH; Bethesda, United States). All efforts were made to minimize animal suffering and to reduce the number of animals used.

*Valeriana officinalis* root powder was obtained from “Farmácia Homeopática Única,” a local manipulation pharmacy. The powder from the pulverized rhizome of the plant was diluted in water (125 mg/ml), heated to 100°C for 10 min, and then centrifuged in the water bath at 672 *g* for 10 min. Then, the aqueous extract was filtered and stored at 4°C. The extract and saline (vehicle) solutions were administered orally by gavage for 15 days (postnatal days 60–74). Each animal (*n* = 8 vehicle-treated and 8 valerian-treated rats) received either a single daily gavage of 2 ml/kg vehicle or *V. officinalis* extract at a dose of 250 mg/kg/d in a volume of 2 ml/kg. The doses of *V. officinalis* were based on the daily recommended dose for an adult human, which is 3,060 mg (as suggested on the bottle of a commercially available product).

Rotenone (obtained commercially from Sigma, St. Louis, MO, United States) was suspended in 1% Tween-80 solution in water and dispersed by sonication. Administration of rotenone consisted of daily subcutaneous injections at a dose of 10 mg/kg/d for 7 days (postnatal days 68–74) in a volume of 1 ml/kg (*n* = 7 rats). This dose was chosen based on previous studies in rodents (Dodiya et al., 2018; Perez-Pardo et al., 2018). The solution was freshly prepared every day. The control group (*n* = 9) was treated with the vehicle (1% Tween-80 solution) in a similar volume and time schedule. In the group that received *V. officinalis* and rotenone treatment simultaneously (*n* = 5), the subcutaneous rotenone injection occurred over the last 7 days of the 15-day period of *V. officinalis* administration.

The electrophysiological recording of CSD occurred on postnatal day 75–80. The animals were anesthetized with a mixture of 1 g/kg urethane plus 40 mg/kg chloralose via intraperitoneal injection. Three trephine holes (2–3 mm in diameter, aligned in the frontal-to-occipital direction, and parallel to the midline) were drilled into the right side of the skull. The CSD-eliciting stimulus (a 1–2 mm diameter cotton ball saturated with a 2% KCl solution) was applied for 1 min to the frontal hole. This stimulus, which elicited a single CSD episode, was repeated at 20-min intervals over the 4 h of the recording session. Two recording electrodes (Ag-AgCl type) were placed on the two parietal holes. These electrodes were glued one-to-the-other with cyanoacrylate glue, and presented a fixed interelectrode distance. A third Ag-AgCl electrode was positioned on the nasal bones and was used as common reference electrode (Lima et al., 2014). We calculated the CSD velocity of propagation dividing the interelectrode distance by the time required for a CSD wave to pass that distance. Rectal temperature was maintained at 37 ± 1°C with the help of a heating blanket. At the end of the recording session, the animals were euthanized with an overdose of anesthetic.

### *In vitro* Experiments

The C6 rat glioma cell culture was prepared following previously described procedures (Goswami et al., 2015). Briefly, cells were placed on plastic plates (10 cm diameter) with Dulbecco’s modified Eagle’s medium (DMEM) supplemented with 1 mM pyruvic acid, 2 mM L-(+)-glutamine, 44 mM NaHCO_3_, 10% fetal bovine serum, 100 IU/ml penicillin, and 100 μg/ml streptomycin. The culture medium was renewed every other day. Cultures were maintained at 37°C until complete confluence (approximately 2–4 days), then they were trypsinized and placed on 96-well plates (3.1 × 10^4^ cells/cm^2^). In a triplicate experiment, cells were treated with rotenone concentrations ranging from 1 to 200 nM or with 0.5% DMSO (vehicle) for 48 h to determine the cytotoxic concentration of rotenone that killed 50% of cells (EC_50_). This concentration was used to investigate the protective effect of *V. officinalis*. The root powder of *V. officinalis* was successively extracted three times for 3 days in petroleum ether, dichloromethane, and methanol. The methanol extract was used in this work containing between 0.2 μg/ml and 153 μg/ml of the *V. officinalis* powder in a duplicate experiment. We evaluated cell viability by using the dye 3-(4,5-dimethylthiazol-2-yl)-2,5-diphenyltetrazolium bromide (MTT assay; Hansen et al., 1989). Briefly, 1 mg/ml MTT was added to each well, and after a 2 h-incubation at 37°C, 100 μl of lysis buffer [20% (w/v) sodium dodecyl sulfate (SDS) in 50% (v/v) *N,N*-dimethylformamide, pH 4.7] was added to each well. The optical density, which is proportional to the number of viable cells, was evaluated with a microplate reader at 595 nm. The cell morphology was examined by phase-contrast microscopy.

### Statistical Analysis

For the *in vivo* experiments, statistical differences were analyzed using Sigmastat^®^ statistical software (version 3.5, San Jose, CA, United States). Intergroup differences were compared using ANOVA. When indicated, ANOVA was followed by a *post hoc* (Holm-Sidak) test. Differences were considered significant when *p* < 0.05.

For the *in vitro* experiments, we used GraphPad Prism version 5.00 for Windows (GraphPad Software, San Diego, CA, United States). The D’Agostino and Person omnibus normality test was used to identify whether data presented normal distribution. Data were expressed as the mean ± SEM or median and ranges according to the distribution. Additionally, according to the distribution, parametric or nonparametric statistic tests were chosen, as indicated in the figure legends. Probability values of *p* < 0.05 were accepted as indications of statistically significant differences. Nonlinear regressions were carried out to fit cell viability data in order to determine the rotenone concentration that killed 50% of cells (EC_50_).

## Results

### *In vivo* Experiments

**FIGURE 1:**
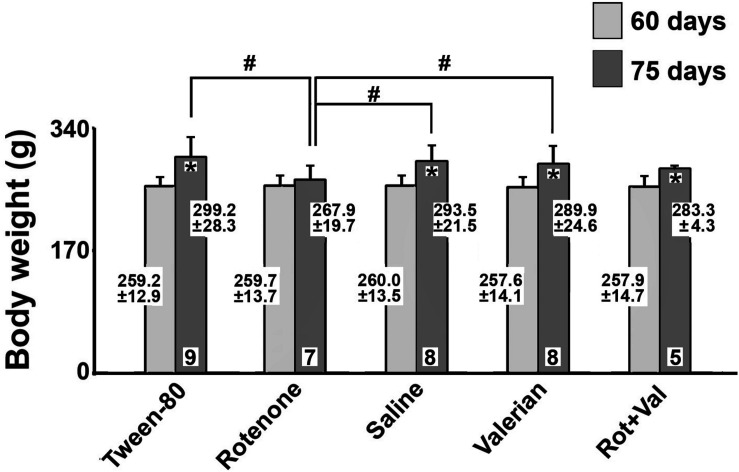
Body weights (mean ± standard deviation; values are given at the bars) of Wistar rats treated via subcutaneous injection of Tween-80 or rotenone or via gavage with saline solution, valerian, or with rotenone + valerian (Rot+Val). Body weights were measured on postnatal day 60 (beginning of experiment), and postnatal day 75 (end of experiment). *p < 0.05 compared with the corresponding 60 day-value. ^#^*p* < 0.05 as indicated by the lines (one-way ANOVA followed by the Holm-Sidak test).

The body weights of the rats in the five groups are presented in Figure 1. At the beginning of the treatment (postnatal day 60), body weights were comparable in the five groups (mean weights ranging from 257.6 ± 14.1 *g* to 260.0 ± 13.5 *g*). At the end of the treatment period (postnatal day 75), the rotenone-treated group had a lower mean body weight (267.9 ± 19.7 *g*) when compared with the Tween-80 group (299.2 ± 28.3 *g*), the saline group (293.5 ± 21.5 *g*), and the *V. officinalis* group (289.9 ± 24.6 *g*; *p* < 0.05).

#### CSD Propagation Velocity

**FIGURE 2:**
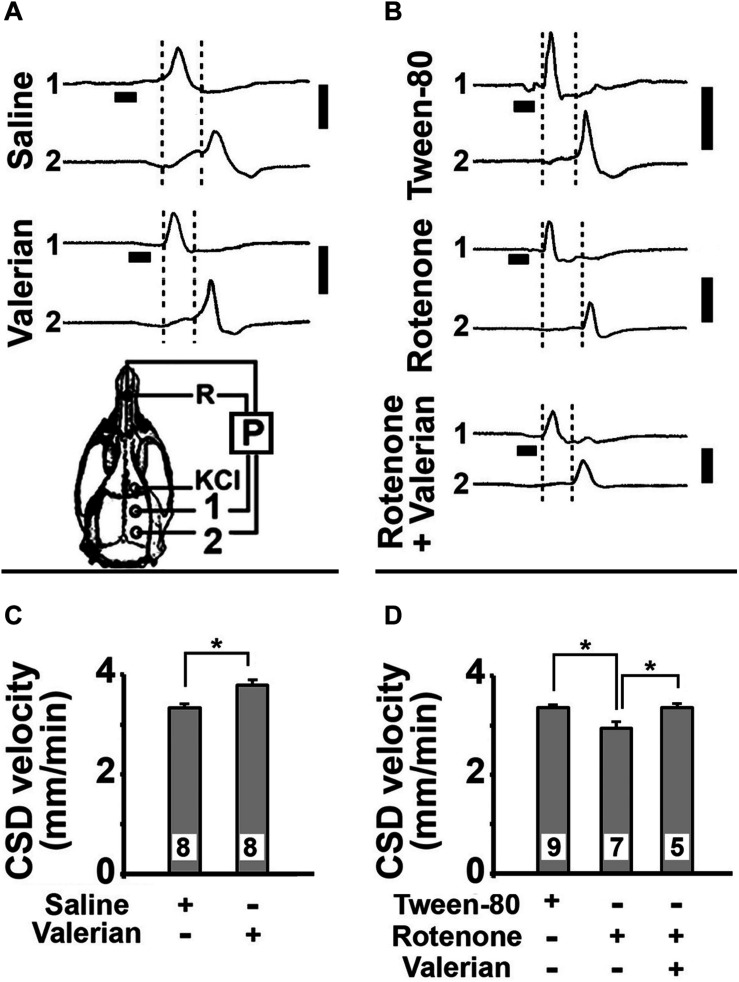
**(A,B)** Electrophysiological recordings (slow potential changes, P) of CSD in five rats that were treated per gavage with *V. officinalis* (250 mg/kg/d) or saline solution for 15 days, or with s.c. injections of Tween-80 (control group) or rotenone (10 mg/kg/d) for 7 days, or treated with s.c. injections of rotenone plus gavage with *V. officinalis*. The vertical solid bars at the right of the traces indicate 10 mV (negative upwards). The horizontal bars under the traces from the recording point 1 indicate the time (1 min) of application of the CSD-eliciting stimulus (a 2 mm diameter cotton ball soaked with 2% KCl solution) on the intact dura mater. Once elicited in the frontal cortex, CSD propagated and was recorded by the two cortical electrodes located at the parietal cortex (skull diagram, points 1 and 2). A third electrode of the same type was placed on the nasal bones and served as a common reference (R) for the recording electrodes. The vertical dashed lines delimited the latency for the CSD wave to cross the interelectrode distance. When compared with the corresponding controls, the latencies in the *V. officinalis* group and in the rotenone group were, respectively, shorter and longer. **(C,D)** CSD velocity of propagation in the 75–80-day-old rats of the five groups described in **(A,B)**. Values are presented as the mean ± standard deviation of the 12 CSD measurements per rat, along the 4 h recording period, with the number of animals given at the bottom part of the bars. Asterisks indicate intergroup differences (*p* < 0.05; ANOVA plus the Holm-Sidak test).

Topical application of 2% KCl for 1 min on the frontal cortex initiated CSD that was recorded at two points on the parietal surface. In parts A and B of Figure 2 we show examples of CSD recordings in five rats that were treated with saline, valerian, Tween-80%, rotenone, or valerian plus rotenone. After a KCl-elicited CSD initiates, the cerebral cortex activity recovers within a few minutes (usually less than 10 min; Leão, 1944). Considering this recovery time, we established 20 min as time-interval to repeat the KCl stimulation over the 4 h of the recording session.

Figures 2C,D presents the mean CSD propagation velocities in the five groups of rats that were studied *in vivo*. ANOVA revealed a significant difference between the treatment groups [F(4,32) = 75.98; *p* < 0.001]. The *post hoc* (Holm-Sidak) test revealed that CSD velocity was significantly higher in the valerian-treated group (3.81 ± 0.10 mm/min) compared to the saline-treated group (3.35 ± 0.08 mm/min; *p* < 0.005). The rotenone-treated animals displayed lower CSD velocities (2.96 ± 0.14 mm/min) in comparison to the Tween-80-treated controls (3.37 ± 0.06 mm/min; *p* < 0.007). The group treated with rotenone plus valerian displayed a mean CSD velocity (3.38 ± 0.07 mm/min) that was similar to the controls (saline: 3.35 ± 0.08 mm/min; Tween-80: 3.37 ± 0.06 mm/min).

### *In vitro* Experiments

#### The Extract of *V. officinalis* Protected Cell Cultures Against Rotenone-Induced Cytotoxicity

**FIGURE 3:**
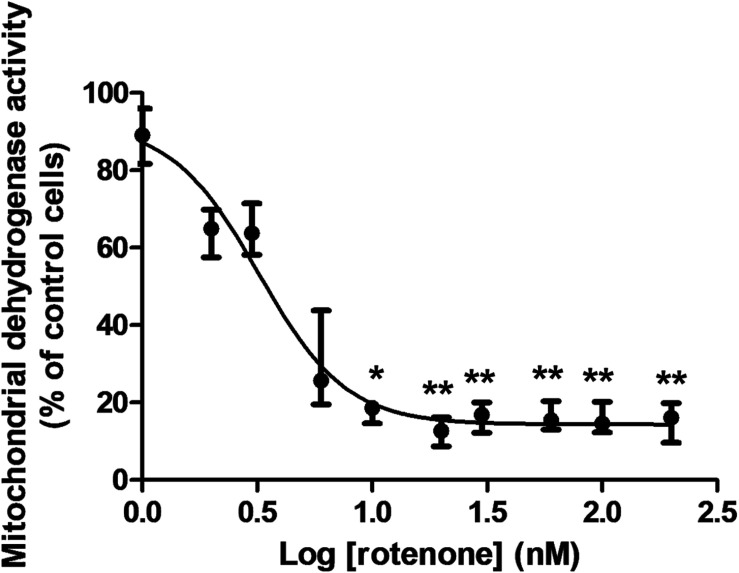
Cytotoxic effect of rotenone to rat glioma C6 cells after 48 h measured by the MTT assay. The graph represents the experiment corresponding to the median EC_50_, which was 3.5 nM. Since the data did not follow a normal distribution, they were represented by medians and ranges. Cell viability corresponds to the mitochondrial dehydrogenase activity measured at 595 nm. Data were normalized to the median absorbance of the control cells treated only with 0.5% DMSO (Median: 1.041; Range: 1.026–1.237; *n* = 8), which was considered a 100% cell viability. Data analyzed by the Kruskal–Wallis test showed a significant effect for rotenone concentration (*p* < 0.0001). Groups were compared by Dunn’s multiple comparison test (*n* = 8 for every concentration). Statistical significance: *p < 0.05; **p < 0.0001 compared to control cells.

Rotenone treatment induced a cytotoxic effect to C6 cells after 48 h in a concentration-dependent manner. The median EC_50_ for rotenone was 3.5 nM (Range: 0.4–4.1 nM; *n* = 3). Data from the experiment corresponding to the median value are shown in Figure 3. Data fitted to Equation (below) using nonlinear regression:

$$\text{V}=14.32+\left\{77.96/\left[1+10^{\left(2.28\,\mathrm{Log}\,\text{C}-1.16\right)}\right]\right\};\left({\text{R}}^{2}=0.9606\right),$$

in which V is the percentage of cell viability and C is the rotenone concentration.

**FIGURE 4:**
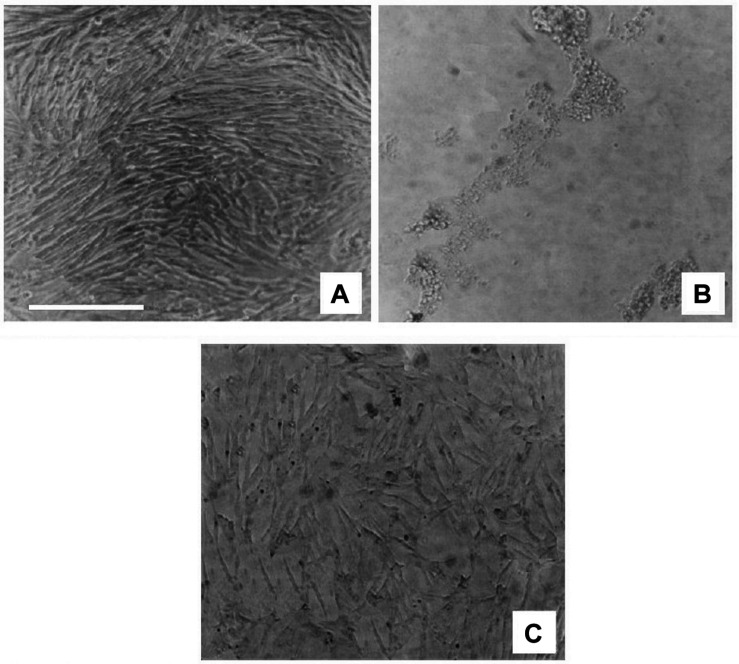
Photomicrographs (phase-contrast microscopy) of C6 rat glioma cell cultures. In **(A)**, we see a culture that received vehicle treatment (DMSO 0.5%). After rotenone application, cell viability decreased significantly **(B)**. The application of 132.1 μg/ml *V. officinalis* to a rotenone-treated culture **(C)** protected the culture against the rotenone toxic effect. Scale bar = 100 μm.

In comparison to the vehicle-treated cultures (treated with 0.5% DMSO; Figure 4A), rotenone treatment induced a concentration-dependent cytotoxic effect on C6 cells after 48 h, as evaluated by decreased cell viability (Figure 4B). Treatment with *V. officinalis* did protect the cells against the toxic action of rotenone (Figure 4C).

**FIGURE 5:**
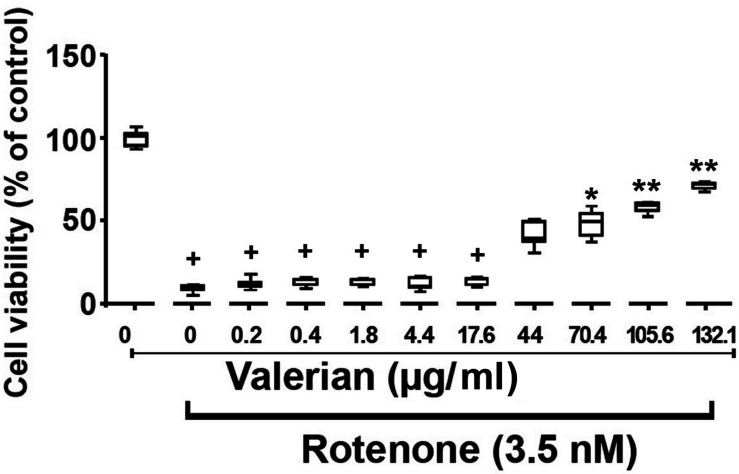
Cytoprotective effect of the methanolic extract of *V. officinalis* root powder against rotenone-induced cytotoxicity in rat glioma C6 cells after 48 h. Cell viability corresponds to the mitochondrial dehydrogenase activity measured by the MTT assay. Data were normalized to the control group, in which cells were treated only with the solvent (0.5% DMSO), which was considered to have 100% cell viability. Since data did not follow a normal distribution, they were represented by their medians and ranges. The group treated with 3.5 nM rotenone was compared with the group treated only with DMSO by the Mann-Whitney test. All groups treated with 3.5 nM rotenone and the methanolic extract of *V. officinalis* root powder between 0.0–132.1 μg/ml were analyzed by the Kruskal–Wallis nonparametric test followed by Dunn’s multiple comparisons test. Statistical significance: ^+^*p* < 0.0005 compared to cells treated only with DMSO; *p < 0.05; **p < 0.0005 compared to cells treated only with 3.5 nM rotenone.

Therefore, the rotenone concentration of 3.5 nM was used for further experiments in order to study the protective effects of the methanolic extract of *V. officinalis* root powder (Figure 5). A significant protective effect of *V. officinalis* against rotenone-induced cytotoxicity was observed. The median of the minimal cytoprotective concentration of *V. officinalis* was 74.8 μg/ml (Range: 40.0–105.6 μg/mL; *n* = 2).

## Discussion

The present results constitute the first evidence that systemic administration of *V. officinalis* and rotenone exert opposite effects on CSD propagation in the rat cortex. Data on this novel electrophysiological action of *V. officinalis* indicate that the treatment with this substance accelerated CSD propagation, whereas rotenone treatment decelerated CSD. Moreover, the combined administration of valerian plus rotenone in the same animal showed that valerian has the ability to protect the brain against the CSD velocity alterations induced by rotenone. Because ovarian hormones seem to influence CSD (Accioly et al., 2012; Accioly and Guedes, 2017), we decided to use only male rats.

The observation that epileptiform EEG waves appeared during CSD (Leão, 1944) originated the speculation that the mechanisms of CSD and epilepsy would be related (Leão, 1944, 1972). During epileptic seizures, redox imbalance occurs, with free radical production and lipid peroxidation; interestingly, all of these processes can cause tissue damage (Tuszkiewicz-Misztal et al., 2000; Ilhan et al., 2006) and can be counteracted by antioxidant compounds that may be present in *V. officinalis* (Rezvani et al., 2010). However, since in the present study redox balance was not investigated, we cannot discuss this potential mechanism as being involved in the plant extract action.

There are some experimental and clinical pieces of evidence suggesting a positive effect of *V. officinalis* against convulsive disorders. In folkloric medicine, *V. officinalis* has been claimed as having an effective anticonvulsant action (Gorji and Khaleghi Ghadiri, 2001; Eadie, 2004). The anticonvulsant action of the aqueous extract of *V. officinalis* was shown by Rezvani et al. (2010) in amygdala-kindled rats using the ethanolic extract of *V. officinalis*, a similar effect reported by Hiller and Zetler (1996), and Petkov and Manolov (1975), respectively, in picrotoxin-induced, and pentylenetetrazole- and strychnine-induced convulsions in mice.

In the *in* vivo part of this study, the effect of an aqueous extract of valerian on CSD velocity was evaluated. Our data suggest that administration of *V. officinalis* was associated with brain changes that resulted in acceleration of CSD propagation. Because no CSD alteration was found in gavage-treated rats (as controls), we can discard the possibility that the CSD effect in the *V. officinalis* group would be produced by the stress of the gavage process, as recently postulated for other compound (Gondim-Silva et al., 2019). This finding confirms previous data indicating that substances with anticonvulsant activity (e.g., dipyrone; Doretto et al., 1998) enhance the CSD velocity (Amaral et al., 2009). Moreover, these data are similar to previous findings showing that low and high doses of the antioxidant vitamin ascorbic acid decelerated and accelerated CSD, respectively, in rats (Mendes-da-Silva et al., 2014, 2018). Taken together, these findings are in accordance with the idea that the CSD, as an interesting phenomenon, can help in extending our knowledge about the involvement of redox imbalance into the pathogenesis of neurological diseases (Guedes et al., 2012) with the possible involvement of astrocytes (Torrente et al., 2014). This possibility shall be investigated in the near future.

Another important finding of the present study was the protective effect of *V. officinalis* on CSD-velocity changes induced by rotenone. Two interpretations can derive from the group treated simultaneously with *V. officinalis*+rotenone (Figures 2B–D): **(a)** the plant extract protects against the rotenone effects on CSD propagation, **or (b)** rotenone inhibits the increment of CSD velocity induced by *V. officinalis* extracts. The first interpretation **(a)** is supported by our *in vitro* outcome (Figures 3–5), showing a protecting action of *V. officinalis* against the rotenone effects on rat C6 glioma cells. Our data on body weight effects (Figure 1) also favor this first interpretation, which is, in addition, coherent with data from others showing several kinds of neural protection that is exerted by *V. officinalis* (Sudati et al., 2013; Becker et al., 2014; Torres-Hernández et al., 2015; Mineo et al., 2017). Further specific investigations are required to test the hypotheses that have been raised to explain the mechanisms, through which *V. officinalis* protects the brain against convulsions. At this stage, we cannot disregard the hypothesis that several mechanisms may operate in producing seizures, depending on the distinct brain regions that are involved (Guedes et al., 1992; Amâncio-dos-Santos et al., 2006).

The underlying mechanisms of the beneficial CNS effects of *V. officinalis* is still a matter of debate. The involvement of the GABAergic system has been postulated based on *in vitro* studies (Mennini et al., 1993). However, mechanisms other than the GABAergic have to be considered. For example, the effects of some components of *V. officinalis* seem to be associated with their antioxidant activities. Indeed, Sudati et al. (2009) showed that *V. officinalis* prevents prooxidant-induced brain oxidative damage. Redox imbalance is associated with cell death that occurs in convulsive states and brain ischemia; interestingly, the treatment with antioxidant agents diminishes this effect (Pestana et al., 2010; Betti et al., 2011).

Since CSD’s are linked to a huge sustained depolarization of the glial membrane potential (Sugaya et al., 1975) the rotenone cytotoxicity to a cell of glial origin was investigated. Rotenone was cytotoxic to rat glioma C6 cells in the present work with an EC_50_ of 3.5 nM after 48 h. This compound acts as an inhibitor of mitochondrial complex I (Nicholls and Budd, 2000). Furthermore, 500 nM rotenone caused apoptosis after 24 h in exposed human dopaminergic SH-SY5Y neuroblastoma cells (Wang et al., 2002). In the cortex exposed to the combination of CSD and the injection of fluorocitrate, which is taken up selectively by astrocytes inhibiting the enzyme aconitase, potentially injured cells expressing the 72-kDa heat shock protein have been observed (Lian and Stringer, 2004). Rotenone is a complex aromatic compound that presents a modified catechol group in its molecular structure. Aromatic compounds such as catechol may be toxic to cells from the central nervous system (Lima et al., 2008). 1,3-Dinitrobenzene, which also induces a metabolic impairment, killed 50% of C6 cells after 36 h at the concentration of 630 μM (Miller et al., 2011). Therefore, rotenone was more cytotoxic than this compound. Rotenone at the concentration of 10 μM killed 50% of the non-dopaminergic neuronal Neuro-2a cells after 4 h (Swarnkar et al., 2012a). Rotenone at 1 μM killed approximately 50% of C6 cells after 24 h (Swarnkar et al., 2012b). Therefore, it seems that rotenone cytotoxicity to C6 cells is concentration- and time-dependant. Rotenone at 10 μM was more cytotoxic to differentiated neuroblastoma SH-SY5Y cells induced by retinoic acid than to undifferentiated ones after 24 h treatment (Jantas et al., 2013). This suggests that dopaminergic neuronal cells are more sensitive to rotenone than non-dopaminergic ones. Furthermore, the infusion of rotenone into the right *substantia nigra* in rats lowered the expression of tyrosine hydroxylase in mid brain after 7 days in a dose-dependent manner, which suggests the damage of dopaminergic neurons (Swarnkar et al., 2013). The compound 1-Methyl-4-phenyl-pyridine (MPP+) is another inhibitor of mitochondrial complex I that killed 50% of wild-type mouse astrocytes in cultures at the approximate concentration of 14 μM after 48 h (Khan et al., 2014). Therefore, rotenone was more cytotoxic to C6 cells than MPP+ was to mouse astrocytes. It seems that rotenone was also more cytotoxic to C6 rat glioma cells than to T98G human glioblastoma cells, since the EC_50_ was 50 μM after 24 h (Cabezas et al., 2015). Glial maturation factor, which is a highly conserved neuroinflammatory acidic protein that is released by damaged cells, decreases the viability of N27 rat dopaminergic neuronal cells in a concentration-dependent manner (Selvakumar et al., 2018). This means that this protein released by rotenone-damaged glial cells may mediate dopaminergic neuronal cell death. The use of rotenone *in vitro* and *in vivo* is a successful neurodegenerative model of Parkinson’s disease due to similarities between the neural rotenone effects and the symptoms of that neurological disorder (Cabezas et al., 2019). Pretreatment with medicinal plants such as *Pistacia atlantica* have demonstrated to be neuroprotective against Parkinson’s disease-inducing neurotoxins, like 6-hydroxydopamine and rotenone (Moeini et al., 2019). Therefore, the present study investigated the neuroprotective potential of *V. officinalis*.

The aqueous extract of *V. officinalis* was not cytotoxic up to 391 μg/ml but it presented an EC_50_ of 2.8 mg/ml to human neuroblastoma SH-SY5Y (Oliveira et al., 2009). Valproic acid, a compound derived from valeric acid, which is produced by *V. officinalis*, can counteract by long-term administration selective alterations of α-synuclein caused by rotenone in rodents (Chateauvieux et al., 2010). Valproate also mitigated the oxidative stress produced by FeCl_3_ in primary cultured rat cerebrocortical cells (Ximenes et al., 2012). Another molecule present in *V. officinalis* is 2*S*-hesperidin, a flavonoid with sedative effects in the central nervous system (Roohbakhsh et al., 2014).

In the present work, the median of the minimal cytoprotective concentration of the methanolic extract of *V. officinalis* against rotenone-induced cytotoxicity to C6 cells (Figure 5) was 74.8 μg/ml. *V. officinalis* was considered as a potential alternative phytodrug against neurodegenerative diseases (Perez-Hernandez et al., 2016). It also presented protective effects in *Drosophila melanogaster* subjects that had been challenged with rotenone (Sudati et al., 2013; Ríos et al., 2016). Another valerian species, *V. amurensis* P. Smirn. ex Kom, increased the cerebral cholinergic function, and decreased apoptosis induced by the amyloid-β peptide (1–40) in mice (Dey et al., 2017). People from the Pikuni-Blackfoot tribe use a mix of *V. officinalis* with *Hypericum perforatum* L. to make a tea with relaxant effects to treat the symptoms of Parkinson’s disease (de Rus Jacquet et al., 2017). The ethnopharmacological use of *V. officinalis* to treat dementia has also been considered (Tewari et al., 2018). *V. officinalis* is among the 12 herbal preparations officially listed by the European Medicines Agency for use as a medicinal product (Forni et al., 2019). Overall, these pieces of evidence allow us to postulate the methanolic extract of *V. officinalis* root powder as a promising product that deserves further fractionation to search compounds that exert medicinal effects. The fact that the composition of the valerian extract remains to be determined does not invalidate the publication of the present data that clearly show the extract’s protective effects. We hope that these data spark the interest of research agencies to fund new projects aiming to isolate and characterize these compounds. Despite the fact that there are currently no effective therapies for Parkinson’s disease and no available treatments to slow down its progression, new drugs (either synthesized or from natural products) as well as non-drug therapeutic options continue to be searched, such as cell transplantation and gene therapies (Jamebozorgi et al., 2019). In both cases (drug and no-drug therapy), pre-clinical *in vitro* and *in vivo* studies, specially using rodent models, are ethically necessary before these strategies can move toward clinical trials and applications, in order to solve some biosecurity aspects. The extrapolation of experimental findings from animals to humans is a complex and challenging process, which requires cautiousness. Despite this, our data globally permit the conclusion that investigation of the brain effects of natural products can benefit from experimental models, such as the two models here employed.

We conclude that the present results suggest the beneficial effect of *V. officinalis* in counteracting the alterations of CSD propagation velocity as well as of C6 rat glioma cytotoxicity induced by rotenone. This beneficial action of *V. officinalis*, which has been documented both *in vivo* and *in vitro*, deserves further pharmacological and molecular exploration aimed at its translational application.

## Data Availability Statement

The raw data supporting the conclusions of this article will be made available by the authors, without undue reservation.

## Ethics Statement

The animal study was reviewed and approved by Ethics Committee for Animal Research of the Federal University of Bahia, Brazil.

## Author Contributions

AA and RG conceived and performed the *in vivo* experiments. AA and RE-B conceived and performed the *in vitro* experiments. IG participated substantially in the conduction of the *in vivo* experiments. AA, RE-B, and RG conducted the statistical analysis and prepared the manuscript. All authors contributed to the article and approved the submitted version.

## Conflict of Interest

The authors declare that the research was conducted in the absence of any commercial or financial relationships that could be construed as a potential conflict of interest.
